# Predictive modelling of the COMATOSE transporter reveals a conserved ligand binding pocket for acyl-CoAs

**DOI:** 10.1038/s41598-026-39225-9

**Published:** 2026-02-25

**Authors:** Foteini Bifsa, Katie Simmons, Stephen P. Muench, Alison Baker

**Affiliations:** 1 School of Biomedical Sciences, University of Leeds, Leeds, LS2 9JT UK; 2 Centre for Plant Sciences and School of Molecular and Cellular Biology, University of Leeds, Leeds, LS2 9JT UK; 3 Astbury Centre for Structural Molecular Biology, University of Leeds, Leeds, LS2 9JT UK

**Keywords:** ABC transporters, ABCD subfamily, COMATOSE (CTS), Plant peroxisomal transporters, Structural modelling, Acyl-CoA binding, Docking simulations, Conservation analysis, Biochemistry, Computational biology and bioinformatics, Structural biology

## Abstract

The ABCD subfamily is comprised of mainly peroxisomal ABC transporter proteins which are found in most eukaryotes. They import a range of substrates of the peroxisomal β oxidation pathway. In plants there is a single peroxisomal ABC transporter AtABCD1 also known as COMATOSE (CTS), PXA1, PED3 or ACN1. Substrates are acyl CoAs and CTS possesses intrinsic acyl CoA thioesterase activity (ACOT), which has subsequently been demonstrated in human and yeast homologues. Even though there is no sequence similarity to any known thioesterases, the CoA moiety is cleaved at some point during transport and most likely released within the peroxisome. While there is a wealth of published biochemical and genetic data on CTS, the precise transport mechanism remains unknown. To gain insights into the CTS transport cycle we have utilised AlphaFold2/3 to produce high confidence models allowing us to explore the apo, post-translocation and post hydrolysis states of the protein, and used the available genetic and biochemical data to validate our models. Docking simulations of these models and C22:0-CoA/ CoA revealed key residues, which we propose are part of the putative binding pocket for the acyl CoAs. The D863/Q864/T867 (DQT) triad likely plays a structural role, while the proximity of S810 to the CoA-binding pocket suggests a potential involvement in substrate recognition or binding. Conservation scores and the alignments of CTS models with the cryoEM determined substrate-bound human homologue structures, supported the key interactors of the pocket which can inform future experiments to understand the transport mechanism of this important protein.

**Supplementary Information:**

The online version contains supplementary material available at 10.1038/s41598-026-39225-9.

## Introduction

Peroxisomes are small, membrane-bound organelles present in nearly all eukaryotic cells, that facilitate a range of important cellular processes depending on the taxa they are found. β oxidation is a near universal metabolic pathway where fatty acids and other lipid-like molecules are broken down and the products are fed into other metabolic pathways. In plants and yeasts, peroxisomes are the exclusive site for β-oxidation, whereas in animals this process occurs in both peroxisomes and mitochondria^1^. A range of transporters are found in different species peroxisomes depending on the needs of the organism^2^. The ABCD (ATP-binding Cassette D) protein subfamily mostly handles the transport of acyl-Coenzyme A (Acyl CoAs) across the membrane, with differing specificity among family members (reviewed in Baker et al.^1^; Paudyal et al.^3^.

These proteins belong to ABC (ATP-binding Cassette) gene superfamily, which is one of the largest found in all sequenced genomes and encodes mostly membrane-bound transporters with a large variety of substrates^4^. A functional ABC transporter consists of two sets of transmembrane domains and two nucleotide binding domains, which may be encoded as a single polypeptide, as half transporters comprising one TMD and one NBD that must dimerise, or as separate proteins^5^. The most useful classification system for ABC transporters is based on the structural homology of their transmembrane domains (TMDs)^6^. Type I-III transporters are exclusively importers (transport into the cytosol), while Types IV and V include a diverse range of exporters (transport out of the cytosol, including into organelles). Type IV transporters include the multidrug resistance protein P-glycoprotein and exporter Sav1866, as well as CTS. Type IV transporters share a typical ‘domain swapped’ architecture in which the TMD of one half/monomer of the protein interacts with the NBD of the other.

In humans, there are three peroxisomal members of the ABCD family, ABCD1-3^1^. Mutations in HsABCD1 -also known as ALDP -which transports very long chain fatty acids (VLCFA) are linked to the neurodegenerative disorder X-linked adrenoleukodystrophy (X-ALD)^7^. HsABCD2, also known as ALDR, functions similarly to HsABCD1 but with a broader substrate specificity^8^, while HsABCD3, also known as PMP70, is essential for the import of branched-chain fatty acids and bile acid intermediates^9^. The human ABCD proteins are expressed as half transporters with the fold pattern of TMD-NBD and come together to form homodimers. There is a fourth transporter in the D subgroup, which unlike the rest, is localised in the lysosome and is involved in cobalamin transport^10^. Yeast species, such as *Saccharomyces cerevisiae*, also possess peroxisomal ABCD transporters, like Pxa1p and Pxa2p, which are involved in the uptake of long-chain fatty acids (LCFAs)^11^. These two proteins are also half transporters, but they dimerise to form a functional heterodimer. In plants the orthologous protein is a full transporter.

In *Arabidopsis thaliana*, Comatose (AtABCD1; from now on CTS) has been identified as the transporter responsible for transporting acyl-CoAs across the peroxisomal membrane. In oilseeds like *A. thaliana,* β-oxidation is essential during seed germination and early seedling development^12–15^. During this critical phase, stored lipids are mobilised and broken down via peroxisomal β-oxidation to yield energy and carbon skeletons. Seedlings lacking CTS are unable to establish without the addition of exogenous sucrose^12^.

CTS was identified by multiple forward genetic screens as well as through reverse genetics^12–14,16^. This highlights not only its importance in supplying substrates for β-oxidation but also the significantly varied compounds it is believed to accommodate within its transmembrane domains. Most evidence of the wide range of substrates is inferred from genetic studies where phenotypic differences are compared between mutants and the wild type. For example, *cts* mutants exhibited reduced jasmonic acid (JA) levels^17^. JA is a member of the jasmonate class of plant hormones that control response to biotic and abiotic stresses, plant growth, plant development and take part in the wound response^18^. Other putative substrates include indole butyric acid (IBA)/IBA-CoA^15^, 2,4-dichlorophenoxybutyric acid^13^ acetate^16^ and cinnamic acid/cinnamoyl-CoA^19^. However, the experimental challenges of purifying this large eukaryotic ABC transporter in active form and lack of commercially available substrates has hindered mechanistic understanding of this important protein.

Protein structure predictive software, like AlphaFold, has been able to aid our understanding of membrane proteins whose structures are difficult to determine experimentally. Traditional methods such as X-ray crystallography and Electron Cryo-Microscopy (CryoEM) often face significant challenges with membrane proteins due to their hydrophobic regions. Additionally, the dynamic and flexible nature of membrane proteins, along with their interactions within the lipid bilayer, further complicate structural determination. AlphaFold2 and 3, developed by DeepMind, have been proven to be very powerful tools in structural biology, utilising advanced deep learning techniques to accurately predict protein structures^20,21^. Given the amino acid sequence as input, it uses multiple sequence alignment to collect evolutionary data, thus identifying conserved regions within the protein. The complex neural network architecture and the ability to predict pairwise distances and bond angles of amino acids, can result in highly accurate 3D models of membrane protein structures, providing insights into their structures and functional properties^22,23^.

In this study, we used AlphaFold2 and AlphaFold3 to model multiple conformational states of the Arabidopsis peroxisomal ABC transporter COMATOSE (CTS), including apo, ATP-bound, and ADP-bound forms. Through structural comparisons, docking simulations with C22:0-CoA and CoA, and conservation analyses, we identified a putative CoA-binding pocket formed by conserved polar residues within the TMDs. Structural alignment with the human ABCD1 transporter and mapping of known CTS mutants onto the models further supported the functional relevance of key residues. Our analyses offer hypothesis-generating insights into substrate recognition and transport by CTS and establish a structural framework that should be interpreted as a guide for future experimental investigation.

## Results

### AlphaFold2/3 predicts different states of CTS

**Fig. 1:**
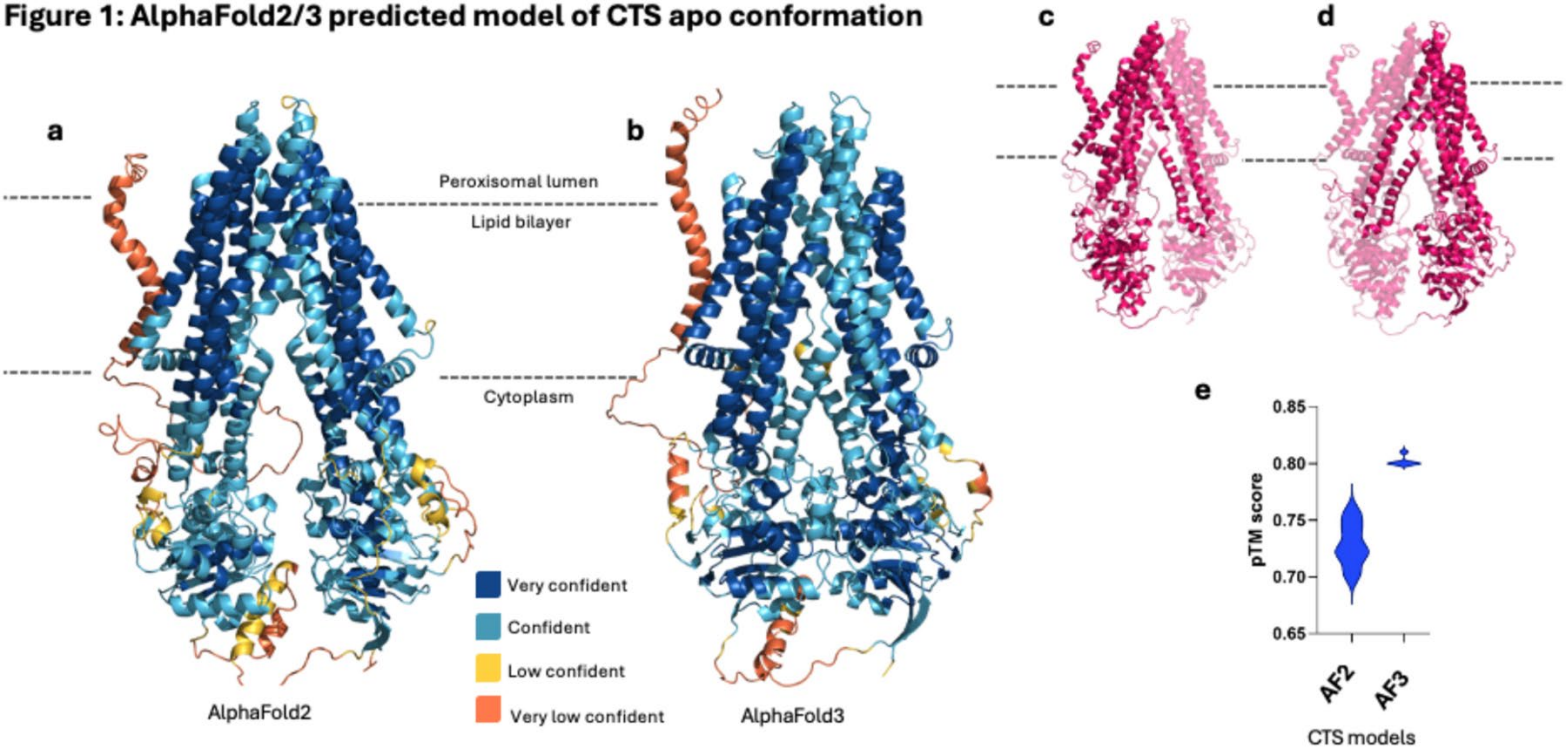
(**a**, **b**) AlphaFold2 (left) and Alphafold3 (right) maps showing prediction scores in dark blue as very high (pLDDT > 90), light blue as confident (pLDDT > 70), yellow as low (pLDDT > 50) and orange as very low (pLDDT < 50). (**c**, **d**) CTS prediction models highlighting the TMD-NBD-TMD-NBD fold. (**e**) Violin plot depicting the summary pTM scores for the each of the five models produced by AlphaFold 2 and 3.

AlphaFold2 (AF2) and AlphaFold3 (AF3) were utilised to generate prediction models for CTS without any co-factors or substrates (apo form). Both AF2 and AF3 produced models with high confidence scores, which indicated they were reliable predictions (Fig. 1a,b). The models revealed CTS adopted a similar fold to other type IV ABC exporters (Fig. 1c,d). The TMDs consisted of 12 helices, 6 for each domain, like the other published structures of the ABCD family, HsABCD1^24–28^ HsABCD3^29^ and HsABCD4^30^. Helices 4–5 for each TMD were seen to interact with the opposite NBD in a domain-swapped architecture. The areas with the highest confidence were the core TM helices and NBDs, while the area with the least confidence was found at the N-terminal end consisting of the first 80 residues which is predicted to be a helix spanning the membrane followed by a large, disordered region (visible in orange).

The generated predicted Template Modelling (pTM) scores of the all the apo structures ranged from 0.70 to 0.80 (Fig. 1e) with a pTM score above 0.5 indicating the overall predicted fold for the protein is likely to resemble the true structure. The reported confidence of prediction as well as the great similarity of models produced by AF2 and AF3, meant both could be used interchangeably to model the apo structure of CTS. Additionally, alignment of the five models produced for both AF2 and AF3 (Fig S1, Fig S2) showed very similar conformations with minimal differences between the helices. The AF3 model appeared more constricted even though the topology remained the same. This is because AF3 handles disordered regions differently to AF2 and thus, the latter produces more “relaxed” structures of proteins^20,21^. The mean RMSD (Root Mean Square Deviation) scores were 1.6 Å for the AF2 models and 1.0 Å for the AF3 models, highlighting the consistency within the predicted models of each output.

**Fig. 2:**
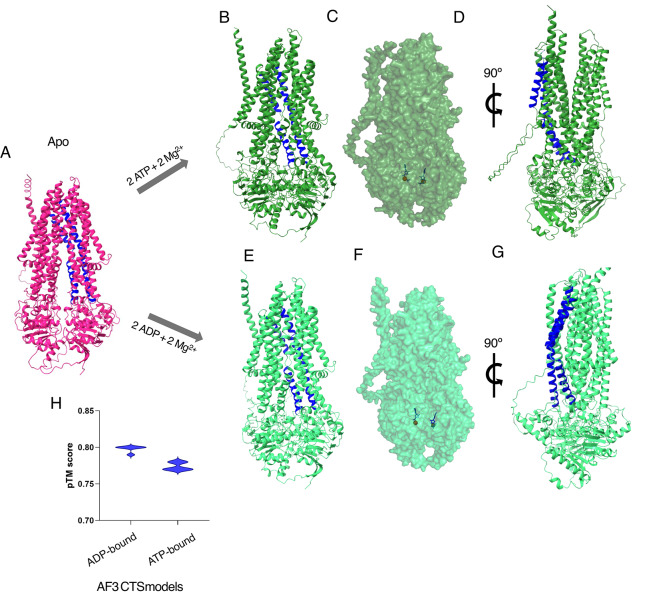
(**a**)Model of CTS in the predicted apo form (hot pink) by AlphaFold3 (AF3) and helices 5 and 6 coloured blue for clarity. (**b–d**) AF3 cartoon and (**d**) surface prediction maps of CTS with two ATP molecules (blue) and two magnesium ions (red) bound. (**e**–**g**) AF3 cartoon and surface prediction maps of CTS with two ADP molecules (blue) and two magnesium ions (red) bound. (**h**) Violin plot depicting the summary pTM scores for the ATP and ADP-bound models produced by AlphaFold3.

AlphaFold3 has been expanded to allow users the ability to model multiple proteins as well as the addition of ligands and ions. We employed AF3 to generate models of CTS either with two ATP or ADP molecules, and two magnesium ions. Interestingly, the addition of the nucleotides was enough to produce conformationally different structures when compared to the one without any additional ligands (apo). The AF3 apo model (Fig. 2a) when compared to the AF3 ATP-bound model produced an RMSD score of 5.2 Å over 1301 Cα (C-alpha carbons). The two NBDs were closer together in a dimerisation state (Fig. 2b,c). The side view of the same model (Fig. 2d) shows the TMDs further apart with a noticeable gap in the inner TMDs. Helices 5 and 6 for each half showed the most movement away from the central axis, while the rest of the helices showed more subtle yet noticeable conformational changes. This opening is seen in many type IV ABC exporters^31,32^ as the central cavity where the substrate binding pocket normally lies, collapses and the substrate is released.

**Fig. 3:**
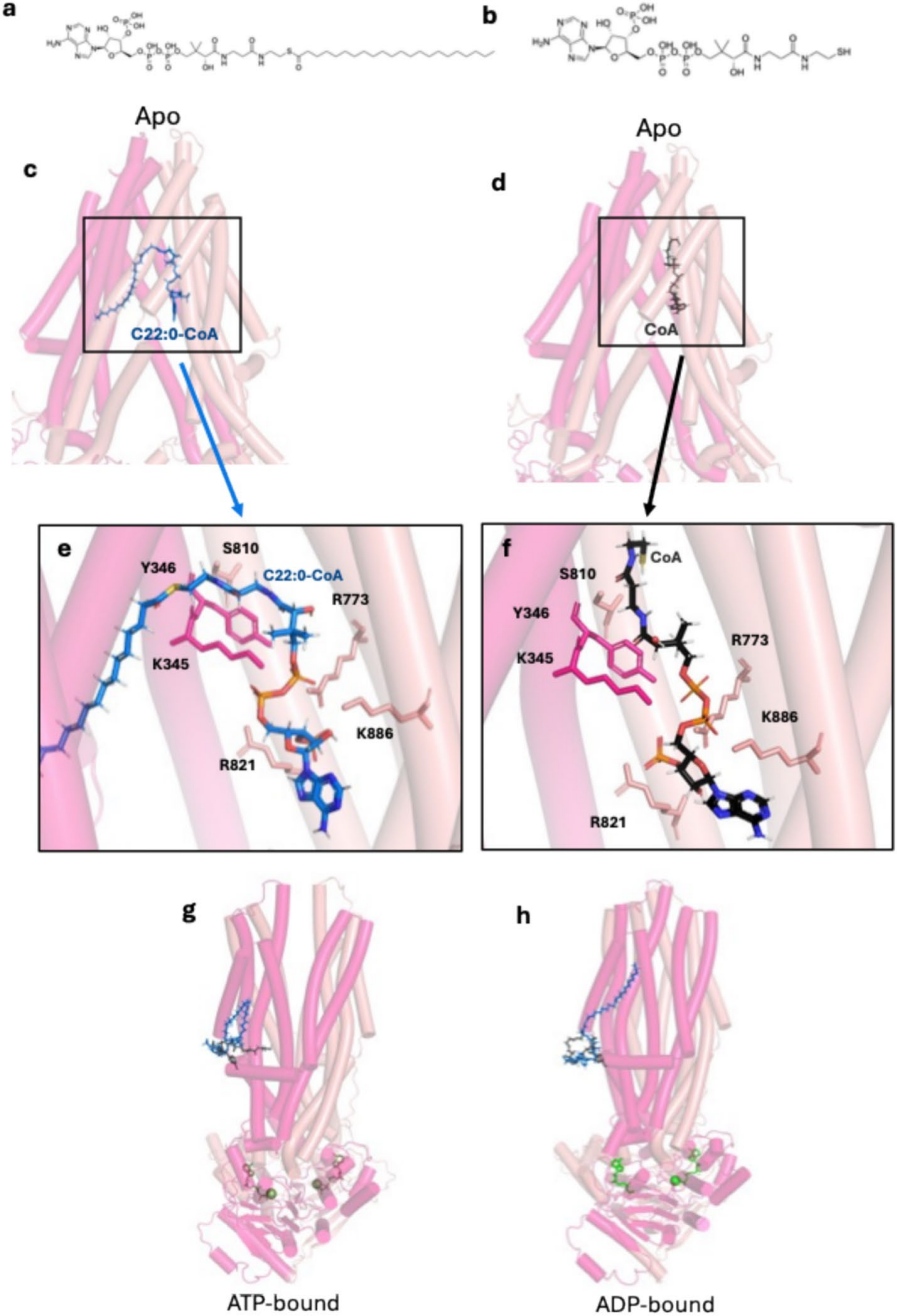
Models of CTS and docking simulations of ligands (**a**) C22:0-CoA in blue and (**b**) CoA in black. (**c**, **d**) CTS structure of AF2 apo model with docked C22:0-CoA in blue and CoA in black, which are both found within the TMDs of the protein. (**e**, **f**) Zoomed in view of the CoA surrounded by residues K345, K886, R773, R821, S810 and Y346. Side view of representative structures of (**g**) ATP-bound and (**h**) ADP-bound models docked with C22:CoA in blue and CoA in black which are both outside the putative binding pocket.

On the other hand, when CTS was modelled with ADP and magnesium ions (Fig. 2e,f) the output was considerably different to the other models. The ADP-bound structure when aligned to the AF3 apo had an RMSD score of 4.2 Å over 1281 Cα. While looking at the side views of the two nucleotide-bound models (Fig. 2d,g) the biggest change was seen in the TMDs while the NBDs were still together. The ADP-bound model did not have the observable gap in the TMDs but rather adopted a post-hydrolysis structure where the transporter’s binding pocket reforms^33,34^ and the protein prepares for another cycle to begin. The pTM scores generated of the all the ATP- and ADP-bound structures ranged from 0.77 to 0.80 Å (Fig. 3h). Additionally, aligning the CTS ATP-bound model to experimentally determined ATP-bound structures of hsABCD1 (PDB codes 7SHM and 7X0Z) produced RMSD scores of 1.2 Å over 821 Cα and 1.1 Å over 834 Cα, respectively. There are currently no published structures of the ABCD family with ADP bound, however, comparing the CTS ADP-bound model to structures 7SHM and 7X0Z, the RMSD scores were 1.1 Å over 784 Cα and over 798 Cα respectively. Evidently, simply adding the nucleotides was enough to produce conformationally different states which can be used to interpret previously published functional studies.

### Docking reveals key residues in putative binding pocket

The three modelled states of CTS were used for docking with ligands C22:0-CoA and CoA only. C22:0-CoA has been shown to be a substrate for both CTS and HsABCD1^35,36^ and thus was a good candidate to be used as a docking substrate. Glide was used to model acyl-CoA binding because it is specifically optimised for docking, whereas AlphaFold3 was employed only to model the apo and ATP/ADP-bound conformations. The docking software struggled to dock the flexible fatty acid tail as the main interactions are hydrophobic. Hence, even though CoA alone does not stimulate ATPase activity of CTS^35^ it was used alone to remove the hindrance imposed from fatty acid tail.

The docking simulations of the AF2 apo model with C22:0-CoA and CoA alone showed both localised at the same site. Figure 3a–c shows representative structures of the apo form, highlighting the CoA moiety with and without the acyl chain. This site is found within a pocket of hydrophilic residues which is comprised of arginine and lysine residues as well as other charged amino acids. The adenine ring of the CoA for both simulations is stabilised by residues K886, R773 and R821 which are within 3.5 Å of the ligand. Expanding the distance to 5 Å, more residues contribute to the overall stability of the CoA, namely Y346, K345 and S810 (Fig. 3e,f). The flexible fatty acid chain can adopt many different orientations within the TMDs; the top half of the transporter is lined with hydrophobic residues which can easily accommodate the varying sizes of the acyl groups of the CoA esters which CTS is able to transport. For clarity, the full set of ligand-residue interactions for both docking runs is illustrated in Supplementary Fig. 3. Additionally, the AF2 apo docking model with C22:0-CoA was also used to evaluate the impact of residue substitutions in silico by analysing the loss of specific interactions between the residues and substrate. There was loss of specific hydrogen bonding interactions when the mutants S810A, R773A, K345A, Y346A, R821A, and K886A were introduced suggesting a loss of substrate-binding affinity would be seen were these mutants introduced in vitro.

Intriguingly, when docking was performed using the ADP-bound and ATP-bound conformations of CTS (Fig. 3g,h), the substrates could not be accommodated within the TMDs. Instead, both C22:0-CoA and CoA were positioned outside the transmembrane region, associating with surface-exposed regions of the protein. This shift in binding location suggests a conformational rearrangement of the TMDs upon nucleotide binding. The shifting could be beneficial as it reduces the affinity of the binding pocket for the substrate which can then be released in the peroxisome. This peripheral placement of the substrates was dismissed because it lacked any hallmarks of a genuine binding site. The poses failed to form coherent clusters, returned generally weaker docking scores, and did not show the same interactions observed in experimental structures or in the apo model where the pocket remains open.

### Conservation scores and structural alignment

To further explore the importance of the identified residues, ConSurf was used to generate evolutionary conservation scores based on the phylogenetic relations between homologous sequences. The calculations were conducted on 1500 hits that sampled the unique sequences. The AF2 apo model was used, as it was automatically implemented by the software. The analysis revealed distinct patterns of conservation when mapped out on the protein model. ConSurf scores show an evolutionary conservation scale from 1 to 9, where 1 indicates highly variable positions and 9 indicates residues that are strongly conserved across homologous sequences. Conserved residues were predominantly located towards the top and bottom of the TMDs. Less conserved regions were found in flexible loops and surface-exposed helices and hence show tolerance to sequence variation. As expected, areas of the NBDs with important sequences such as the Walker A/B motifs and the coupling helices were highly conserved.

**Fig. 4:**
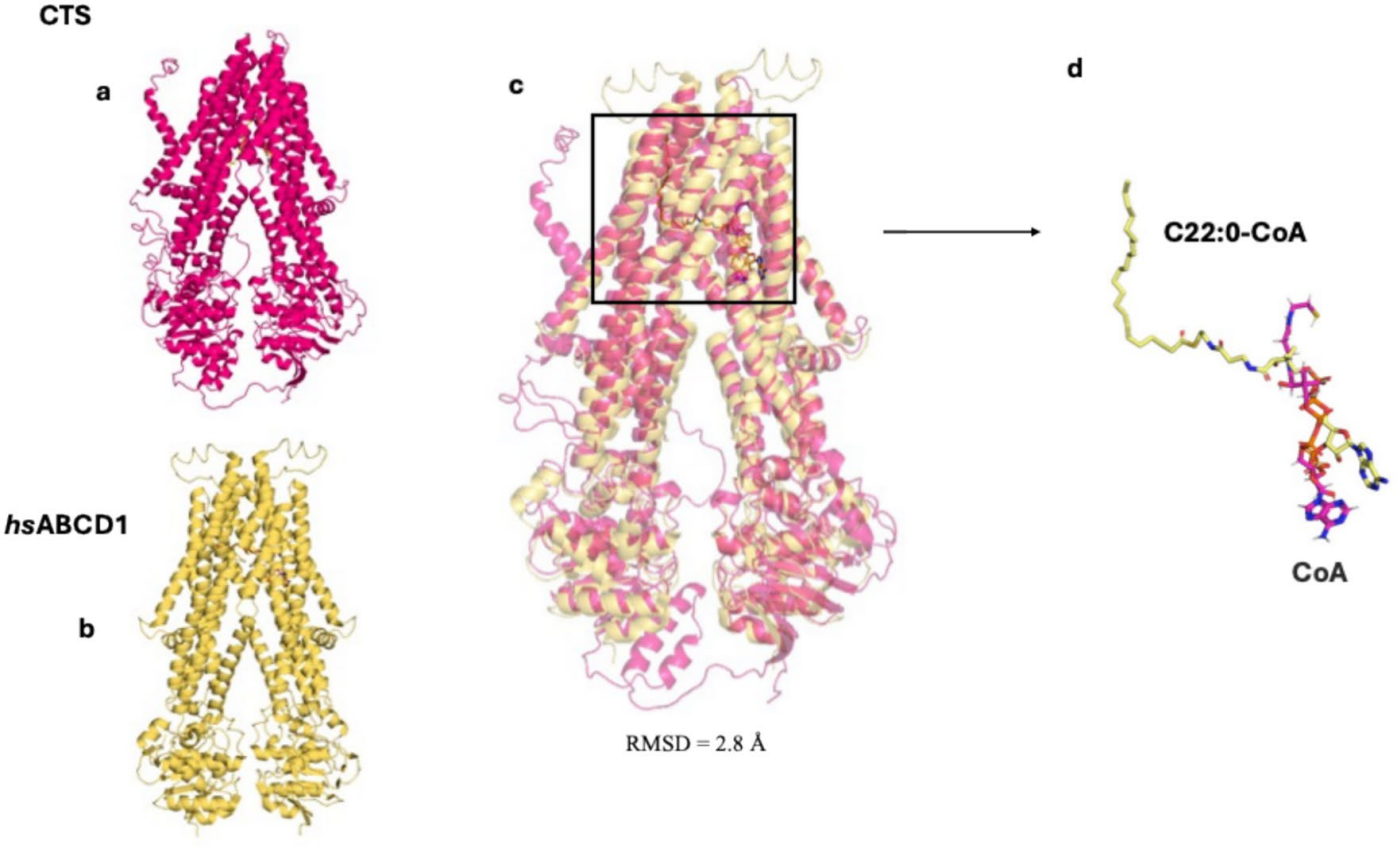
Structural alignment of CTS docked with CoA and HsABCD1 with substrate C22:0-CoA (7VZB). (**a**) CTS apo model superimposed to the (**b**) HsABCD1 with RMSD score of 2.8 Å. (**c**, **d**) The location of the CoA of each structure is similar indicating a conserved CoA binding pocket.

Structural alignment of the CTS apo model (Fig. 4a) and the cryoEM determined structure of the HsABCD1 protein (Fig. 4b) in complex with a substrate^28^, corroborated the likely involvement of the identified CTS residues in binding of the CoA. The human substrate-bound structure was determined with C22:0-CoA (from now on 7VZB). The topologies and arrangement of domains of the human structure and CTS were very similar. (Fig. 4c,d). Superimposing the ConSurf AF2 model and 7ZVB, generated RMSD scores of 2.8 Å over 959 Cα. The alignment indicated the location of the CoA from the 7ZVB structure is the same as the one predicted on CTS from the docking simulations. The residues surrounding the CoA moiety of CTS are the similar in the human structure. This is further confirmed from the scores as most residues appear to be highly conserved. Residues, K345 and R821 had a score of 6, while Y346, R773, S810 and K886 had a score of 8. Specifically, residue K345 showed moderate conservation, with lysine (K) present in 42% of homologous sequences. Alternative residues included serine (S, 11%) and glycine (G, 10%). Residue Y346 showed moderate conservation, with tyrosine (Y) being the most common (51%), followed by glutamine (Q, 28%). Residue R773 was highly conserved, with arginine (R) present in 81% of homologous sequences. A minority of sequences showed a substitution to glutamic acid (E, 10%), suggesting strong selective pressure to maintain a charged residue at this position. Although residue S810 exhibited variability across homologous sequences -with alanine (A, 35%), isoleucine (I, 17%), methionine (M, 13%), and serine (S, 9%)- it was still classified as highly conserved by ConSurf. This suggests functional or structural importance. Residue R821 exhibited moderate sequence variability, with lysine (K, 34%), arginine (R, 24%), glutamine (Q, 13%), and serine (S, 12%) represented amongst the homologous sequences. Finally, residue K886 displayed strong conservation, with lysine (K) occurring in 66% of homologous sequences and glutamic acid (E) in 6%.

### Exploring characterised mutants

**Fig. 5:**
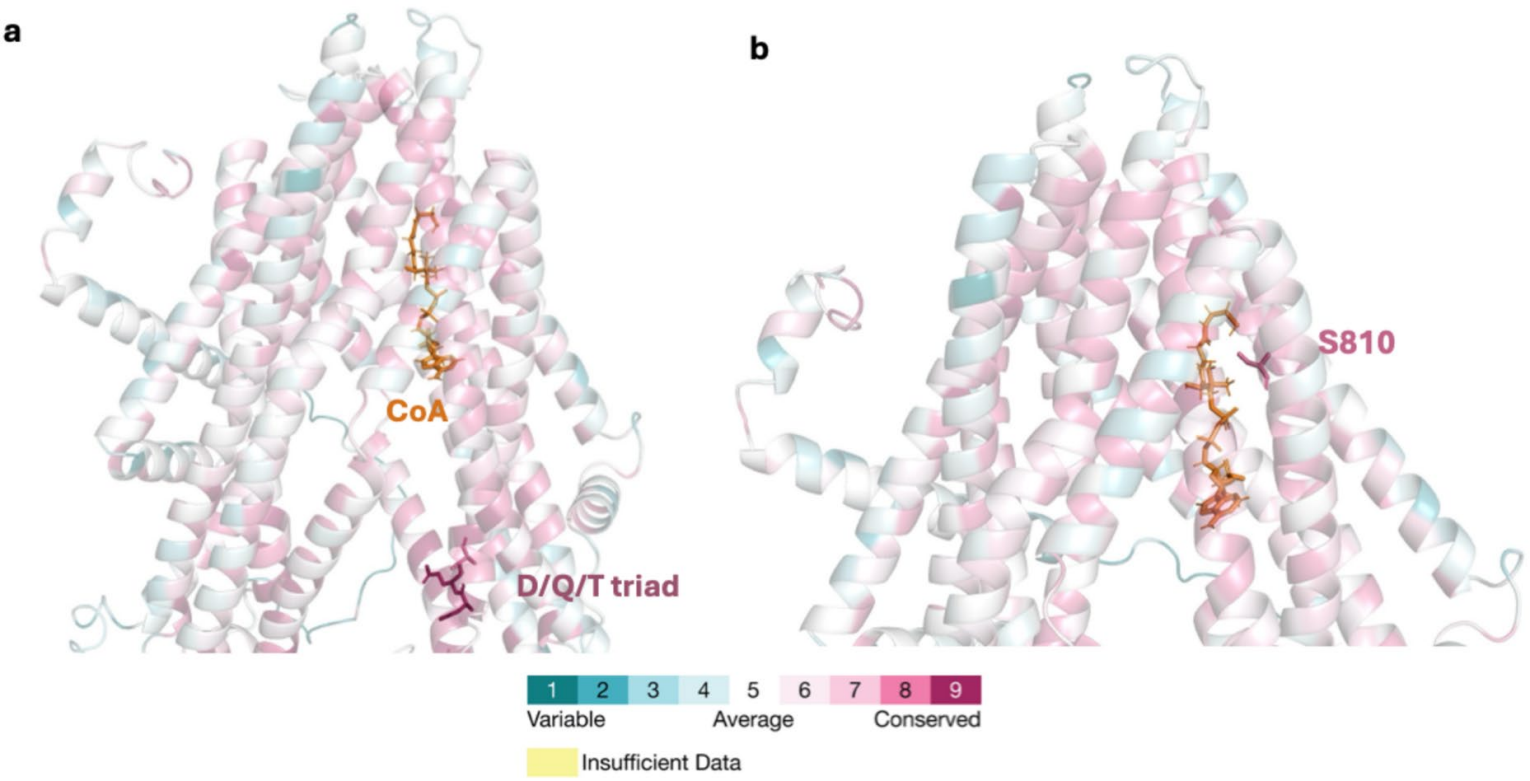
ConSurf AlphaFold2 model coloured according to the conservation scores generated with ConSurf. (**a**) The D/Q/T triad’s (D863, Q864, and T867) distance to docked CoA moiety (orange) is ~ 28 Å. (**b**) Residue S810 shown in relation to the docked CoA (orange) which is within 5 Å.

In the absence of an experimentally determined structure, combining information from new software- like AlphaFold and ConSurf, enables the phenotypic properties of previously characterised mutants to be further examined in a structural context. The AlphaFold models were consistent with the interpretation of previous models (summarised in Table 1). Previously three residues -D863, Q864, and T867 -were identified as potentially forming a catalytic triad for acyl-CoA cleavage or being involved in communicating structural changes^37^. The D/Q/T triad was shown to be highly conserved as ConSurf scores were 9,9 and 8 respectively. Specifically, residue D863 was highly conserved with aspartate (D) bring present in in 90% of homologous sequences, followed by glutamate (E, 6%). Q864 was also highly conserved with glutamine (Q) being present in 94% of sequences and a minority being histidine (H, 3%). T867 was moderately conserved with threonine being present at 65% of sequences. An alternative residue was serine (S, 18%) which also has a hydroxyl group similar to threonine. The conservation scores underline the importance of these residues but so far, their role remained unclear. Comparing the distance between the D/Q/T triad and the putative binding CoA binding pocket, it appears to be ~ 28 Å away from the adenosine ring of the CoA (Fig. 5a). Hence, the triad is located too far to have a catalytic role in the cleavage of the thioester bond of the acyl-CoA.

**Table 1:** Summary of characterised CTS mutants and ConSurf analysis.

Residue	Conserved score	Predicted description	Biochemical result	Reference
D606	9	Exposed Functional (Walker B motif)	D606N in planta doesn’t germinate doesn’t establish	^39^
D863	9	Exposed Functional	D863A in *S. cerevisiae* defective of β-oxidation D863A in peroxisomes retained substrate-stimulated ATPase activity	^41^
E300	9	Exposed Functional	E300D in *A.thaliana* similar phenotype to WT germination	^39^
E953	9	Exposed Functional	E395D in *A.thaliana* were IBA and 2,4-DB sensitive	^39^
K487	9	Exposed Functional (Walker A motif)	K487A in planta doesn’t germinate doesn’t establish K487A in Sf9 membranes: greatly reduced ATPase (not substrate stimulated) and no ATP-dependent ACOT activity	^39^ ^41^
P539	9	Exposed Functional	P539L in planta doesn’t germinate doesn’t establish	^39^
Q864	9	Exposed Functional	Q864A Sf9 membranes: similar ATPase activity to WT but greatly reduced ACOT activity Q864A in *S. cerevisiae* defective of β-oxidation Q864A in peroxisomes retained substrate-stimulated ATPase activity	^41^
S810	8	Buried	S810N in planta germinates(slow) but doesn’t establish S810N *S. cerevisiae* pxa1/pxa2Δ mutant reduced ACOT and unable to support oleate β-oxidation S810N Sf9 membranes: similar ATPase activity to WT but greatly reduced ACOT activity	^39^ ^40^ ^41^
T867	8	Exposed Functional	T867A in *S. cerevisiae* defective of β-oxidation T867A in peroxisomes retained substrate-stimulated ATPase activity	^41^

Another previously well-characterised mutant is S810N^38^. In planta it germinates slowly but does not establish^39^. When the same mutant was expressed in *S. cerevisiae* pxa1/pxa2Δ it showed reduced ACOT and was unable to support oleate β-oxidation^40^. Finally, when expressed in Sf9 insect cell membranes it displayed similar ATPase activity to WT but greatly reduced ACOT activity^41^. This is one of the residues identified being within 5 Å (Fig. 5b) of the CoA binding pocket from the docking simulations and assigned the conservation score of 8. A serine residue near the CoA would be beneficial as the hydroxyl group could become a nucleophile and aid in the cleavage of the thioester bond between the fatty acid and the CoA. The hydroxyl group is oriented towards the binding pocket and is positioned towards the CoA moiety. While the measured distance is within 5 Å, the flexible nature of the fatty acid tail of the acyl-CoA could allow the atoms to come into even closer proximity, supporting the feasibility of this interaction. Other residues of interest from Table 1 are shown on the structural models in Fig S4, but since NBD structures have been available for many years, these have already been discussed in the literature. They are presented for completeness but are not discussed further here, as the model provides no new information about them.

## Discussion

AlphaFold produced high confidence, distinct models for apo, ATP-bound, and ADP-bound which defined 3 different conformational states consistent with experimental data from other related type IV ABC transporters^33,42,43^. Docking simulations independently identified the same binding site for substrate as the experimentally determined HsABCD1 substrate-bound structure^28^ and provide an explanation for how diverse acyl chains attached to CoA could be recognised and transported by CTS. It is also unlikely residues D863 Q864 T867 are part of a catalytic triad, though their conservation and mutant properties make clear they are functionally important, while S810 may be a catalytically important residue in ACOT. The modelling also identifies other residues in the CoA binding site that can be tested by mutagenesis and functional studies in future.

The AF3 predictions for CTS with bound ATP or ADP molecules revealed significant conformational changes compared to the apo state. Considering it was reported AF3 may not be able to model conformational changes when ligands are added and favour a single state^44^, it is notable the addition of ATP and ADP introduces significant changes on the predicted structures of CTS. Comparing the RMSD scores between the ligand-bound and apo states (4.219 Å for ADP-bound and 5.186 Å for ATP-bound) underscores the substantial conformational shifts in the domains that need to occur for substrate translocation. The shifts in the spatial arrangement of TMDs and NBDs upon nucleotide binding have been well-characterised and documented in previous structural studies of ABC transporters^45–47^.

The CoA moiety in the CTS docking simulation localised to the same binding pocket observed in the human structure. This convergence is significant given that the docking was performed independently as the docking grid was centred to encompass the full length of the TMDs. This is suggesting that the identified pocket could be a conserved feature of the ABCD-type transporter family. The residues (K345, K886, R773, R821, S810 and Y346) appear to form a stabilising hydrogen-bonding network for the negatively charged phosphate groups and the ribose of CoA, anchoring the molecule in the pocket. The conservation of the residues suggests the binding of the CoA headgroup is a key determinant in substrate recognition and potentially substrate specificity. The inability to dock C22:0-CoA and CoA alone in the ATP- and ADP-bound models reinforces this interpretation, as these conformations likely correspond to post-binding and post-hydrolysis states, where the central cavity has collapsed or shifted, preventing further substrate entry^48^.

The D863 Q864 T867 conservation scores reinforce the functional importance of these residues^41^ but the distance from the putative substrate binding site suggests its role is structural rather than catalytic. However, this raises the question of what the catalytic residue for the acyl CoA cleavage might be. It has been proposed from the HsABCD1 that cysteine residue(s) are involved in ACOT^49^. The authors propose C39 or C88 being responsible for the ACOT and hypothesise if they could be part of a catalytic triad. There is only one cysteine within the TMDs cavity of CTS -C156- which is equivalent to the S810 residue in the opposite TMD. In order for serine to act as nucleophile, it needs to be activated by a neighbouring base. The possible residues which could create a possible catalytic site near S810 are D341, K345 and Y346. Although tyrosine and lysine are atypical, they could both act as general bases and deprotonate the -OH group. The equivalence of S810 and C156 is not unique in CTS. In fact, most of the amino acids identified earlier have the similar residues in TMD1. C156 is in close proximity to H163, D995 and K999 which are also capable in activating the cysteine to become a nucleophile. Thus, although in this paper we described the potential CoA binding pocket in TMD2, there could be a secondary pocket in TMD1. This mirrored arrangement of residues between TMD1 and TMD2 further supports the idea that CTS functions as a fused heterodimer. The presence of equivalent residues in each half mirrors the functional asymmetry observed in the NBDs. Specifically, it’s been shown NBD1 is essential for transporter activity, as mutations in its conserved Walker A (K487A) and Walker B (D606N) motifs result in a complete loss-of-function phenotype in planta^39^. In contrast, analogous mutations in NBD2 (K1136A and D1276N) do not significantly impair function, indicating that NBD2 is functionally degenerate^39^.

The substrate-bound human ABCD1^25,28^ structures both revealed two separate pockets within each transporter. Perhaps CTS is also able to accommodate two acyl-CoA substrates and possibly bulkier ones like OPDA can only fit one at a time. OPDA is synthesised from linolenic acid (18:3) in the chloroplast, exported via the JASSY transporter^50^ then taken transported into peroxisomes by CTS and converted to JA via β-oxidation^51^. It was found the accumulation of OPDA was more effective at inhibiting WT germination than JA, explaining the phenotype seen in *cts* mutant seeds^52^. The two pockets could resolve the intriguing phenotype of the S810N mutant. We can speculate that even though the mutated residue impairs function, the residues making up the second binding site remain intact which could allow some partial transport activity. This could explain the S810N mutant can germinate, a process inhibited by OPDA^52^ but the mutant couldn’t support oleate β-oxidation which is necessary for seedling establishment^40^.

There are three more structures of HsABCD1 with a substrate present. The structures are reported in inward open conformation and the substrates are C26:0 (PDB:7X07)^26^, C26:0-CoA (7X0T) an E630Q mutant^26^, and C18:1-CoA (PDB: 7SHN)^25^. When these structures are superimposed to AF2 CTS apo model (Fig S5), all three substrates are found close to each other but not at the same location as substrate C22:0-CoA (PDB: 7VZB). These could be considered intermediate stages of the acyl-CoA moving through the TMDs and it could help with the protein recognising the right substrate (acyl-CoA vs FFA).

**Fig. 6:**
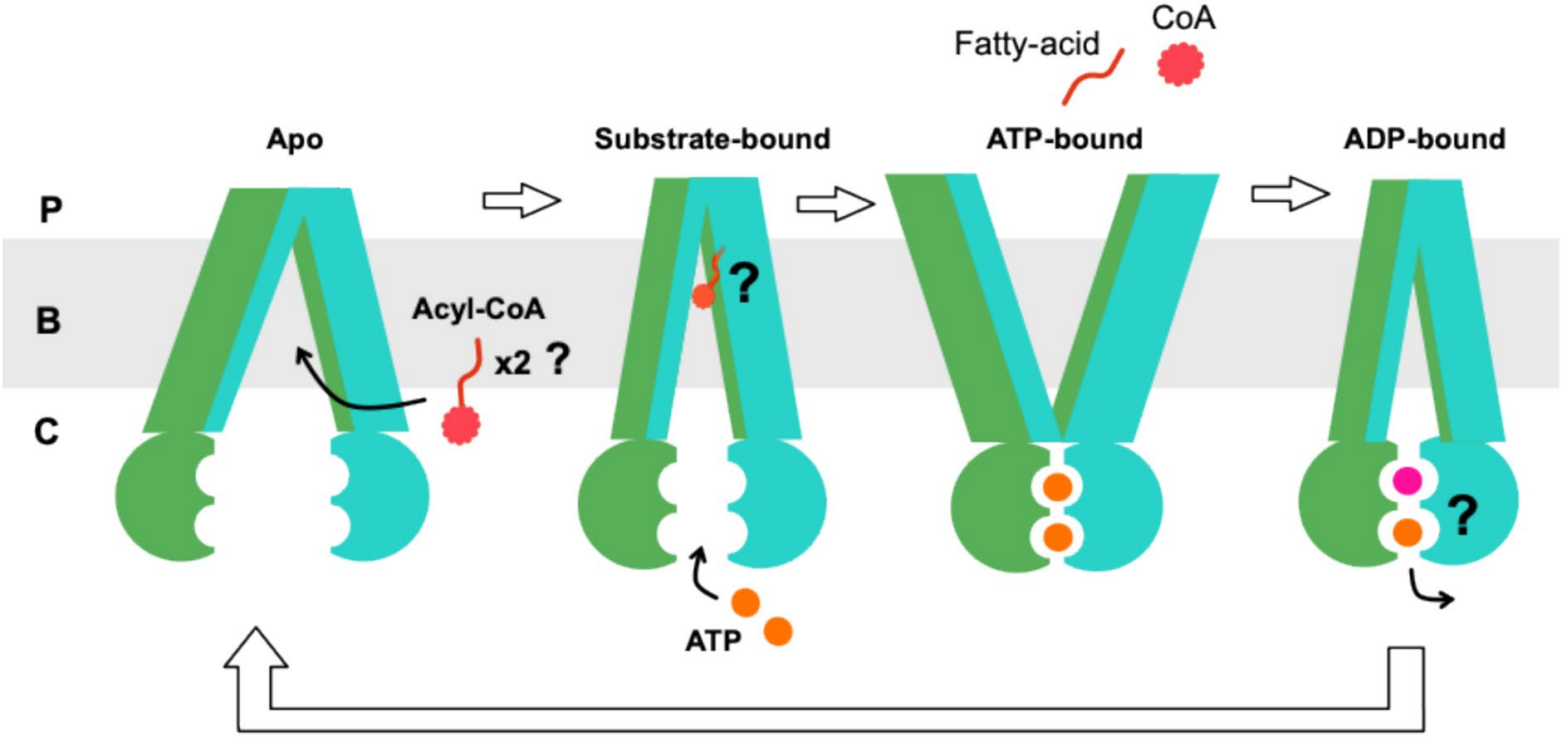
A probable transport cycle for CTS.. The transporter is found on the bilayer (B) between the peroxisomal lumen (P) and the cytosol (C). The transport cycle begins in an inward-facing conformation (apo), with separated nucleotide-binding domains (NBD)s. When acyl-CoA(s)(red) bind to the transmembrane domains (TMDs), conformational changes bring the NBDs closer, promoting ATP (orange) binding and dimerisation. This triggers an outward-facing state, reducing substrate affinity and releasing it into the peroxisome. The CoA is possibly cleaved between the substrate-bound and ATP-bound states. ATP hydrolysis(ADP in pink) then resets CTS back to its resting state, ready for another cycle.

With the current available information, a plausible transport cycle can be proposed for CTS (Fig. 6). CTS adopts an inward-facing conformation (apo), with the two NBDs separated in a rested state. One or more acyl-CoAs may enter laterally through the membrane and bind to the TMDs causing conformational changes. The conformational changes induce an inward-facing state with the NBDs coming closer together (substrate-bound state). This favours ATP binding, and the NBDs come together to dimerise, inducing an outward-facing conformation. This causes changes the binding pocket to have reduced affinity for the substrate and they are released into the peroxisome (ATP-bound). The cleaving of the CoA could occur between the substrate-bound and the ATP-bound states. Finally, the hydrolysis of ATP resets the protein back to the resting state, and it is ready for another transport cycle.

The identification of a conserved CoA-binding site and the proposal of the transport cycle raise important mechanistic questions. It is not known how the substrates enter the binding pocket, if CTS binds one or two acyl-CoAs, and the significance of having a degenerate ATP binding site in the NBDs. Because the AlphaFold algorithms are trained on existing PDB structures, the predicted CTS models are not independent of the broader structural landscape of ABC transporters. Nonetheless, in the absence of an experimental CTS structure, these predictions provide a set of conformations consistent with established features of the ABCD family. The models therefore can be used to generate hypotheses as they highlight a plausible binding pocket and conformational transitions that can now be tested experimentally. Future work should focus on experimentally validating these predictions through site-directed mutagenesis of the binding pocket residues, including the equivalent ones of the other TMD. Mutants can also be used in biochemical assays such as ATPase activity and the ACOT activity assay to identify catalytically important residues and determine whether CTS accommodates one or two acyl-CoA molecules per transport cycle.

Overall, the use of advanced structural predictive software like AlphaFold2 and AlphaFold3, docking simulations and looking into homologous proteins can aid the structural characterisation of membrane proteins but also inform future experiments in the absence of experimentally determined structures. As predictive models continue to improve, they will undoubtedly play an increasingly critical role in structural biology, particularly for challenging targets like membrane proteins.

## Methods

### Alphafold2/3

AlhaFold2 and AlphaFold3 were used to generate the predictive models of CTS. Both were utilised in this study as AlphaFold3 handles disordered regions differently to AlphaFold2^20,21^. The full canonical sequence of the wildtype CTS (Uniprot identification number Q94FB9) was the input in each of the runs. AlphaFold2 was used to predict protein structures via the official Google Colab notebook provided by DeepMind^20^. AlphaFold2 models were downloaded from June 2023. Predicted protein structures generated using AlphaFold3 were done without accessing the online AlphaFold Server. Instead, models were obtained by running the AlphaFold3 pipeline locally using the publicly available implementation provided by DeepMind^21^. Additional inputs were ligands: ADP or ATP and both with magnesium ions added. The pTM scores were pooled together from the output of each AlphaFold run and shown in violin plots.

### Alignment and figures

All structural alignments were performed either PyMOL or PyMOL2 (The PyMOL Molecular Graphics System, Version 3.0 Schrödinger, LLC.). The software utilised the BLOSUM62 matrix for the alignment. The RMSD scores for each comparison (after outlier rejection) were also produced by PyMOL. All structural figures were created with PyMOL.

### Docking simulations

These were performed according to methods published in David. J. Wright et al., 2020^53^. Briefly, the region of CTS assigned for docking studies was chosen to encompass the entirety of the TMDs and excluded the NBDs as the latter is location of ATP binding and hydrolysis. A 20 Å clip of the CTS structure around these residues was termed as the receptor for docking studies using Glide (Schrödinger Release 2024–3, Glide, Schrödinger, LLC, New York, NY, 2024)^54,55^. The CTS structure was prepared using the Protein Preparation Wizard in the Maestro Graphical User Interface (GUI). This aimed to remove any steric clashes of amino acid side chains and optimise the position of hydrogen atoms to facilitate docking studies. The receptor grid was generated using Schrödinger software, allowing docking of ligands in a 66 × 66x66 Å grid seen in supplementary Fig. 6. The C22-CoA and CoA ligands were prepared using the LigPrep module (Schrödinger Release 2024–3, Glide, Schrödinger, LLC, New York, NY, 2024) to produce an energy-minimised 3D structure. Docking of C22-CoA and CoA was carried out using the Glide module of Schrödinger software using the XP mode with flexible ligand sampling and biased sampling of torsions for all predefined functional groups. Epik state penalties were added to the docking score. A maximum of 10 poses for the ligand was requested in the output file and post-docking minimisation was carried out.

### ConSurf parameters and MSA alignment generation

ConSurf was used to estimate the evolutionary conservation of residue positions in CTS based on the phylogenetic relations between homologous sequences^56,57^. The sequence was split into two halves (1–672 and 673–1337) and run individually with the same parameters. Homologues were collected from CLEAN_UNIPROT database with search algorithm being HMMER. The E-value cutoff was 0.001 and the number of iterations was 1. The CD-HIT cutoff was 95%, maximal number of final homologues 1500, coverage 60% and minimal sequence identity with the query sequence was 20%^56,57^.

## Supplementary Information

Supplementary Information.

## Data Availability

The datasets are available at (https:/eur03.safelinks.protection.outlook.com/?url=https%3A%2F%2Fdoi.org%2F10.5518%2F1759&data=05%7C02%7Cbsfb%40leeds.ac.uk%7C17fbf76040b54ec6e9b108ddfc4aad8a%7Cbdeaeda8c81d45ce863e5232a.

## Abbreviations

CTS: Comatose,
IAA: Indole­3­acetic acid
JA: Jasmonic acid
FFA: Free fatty acid
CoA: Coenzyme A
ACOT: Acyl CoA thioesterase activity
cryoEM: Electron cryo-microscopy
ATP: Adenosione triophosphate
ADP: Adenosine diphosphate
AF2: AlphaFold2
AF3: AlphaFold3
ABC: ATP-binding cassette
TMD: Transmembrane domain
NBD: Nucleotide binding domain
X-ALD: X-linked adrenoleukodystrophy
IBA: Indole butyric acid
pTM: Predicted template modelling
OPDA: 12-Oxo-phytodienoic acid
VLCFA: Very long chain fatty acid
TM: Transmembrane
