# Supplementary material for: Predictive modelling of the COMATOSE transporter reveals a conserved ligand binding pocket for acyl-CoAs
Source: Sci Rep. 2026 Feb 25;16:10423. doi: 10.1038/s41598-026-39225-9 (PMC13031526; doi:10.1038/s41598-026-39225-9)
Supplement: Supplementary file 1 — Supplementary Information. [file 41598_2026_39225_MOESM1_ESM.docx]

**Supplementary figures**


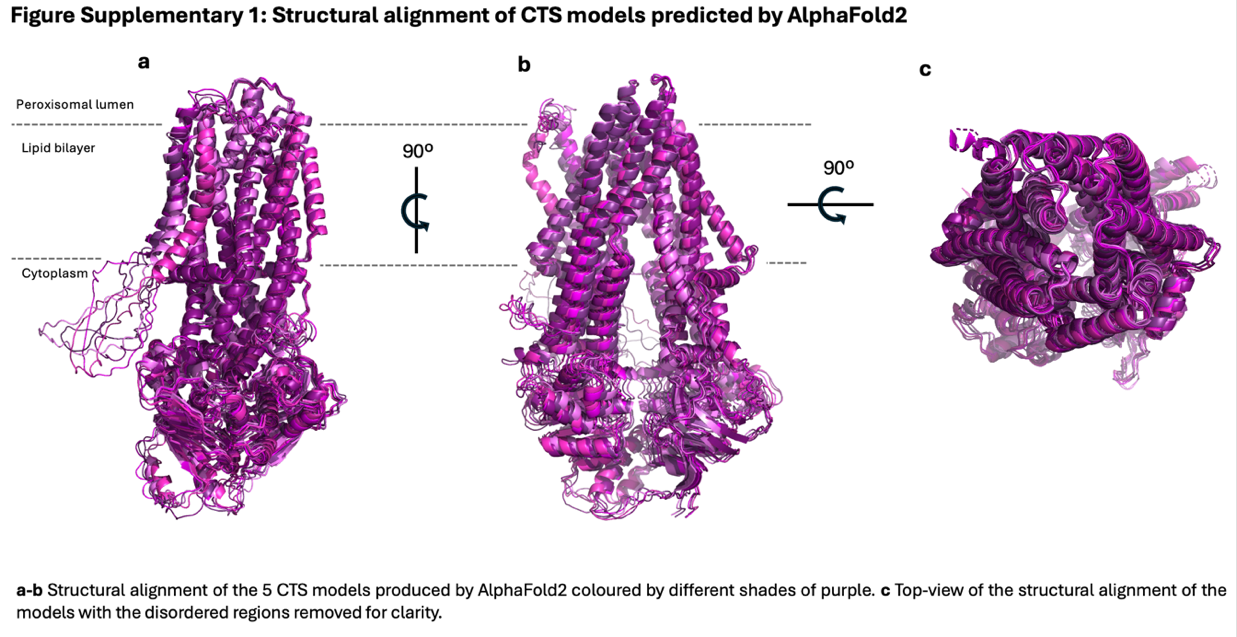


***S. Fig 1. a-b*** *Structural alignment of the 5 CTS models produced by AlphaFold2 coloured by different shades of purple.* ***c*** *Top-view of the structural alignment of the models with the disordered*

*
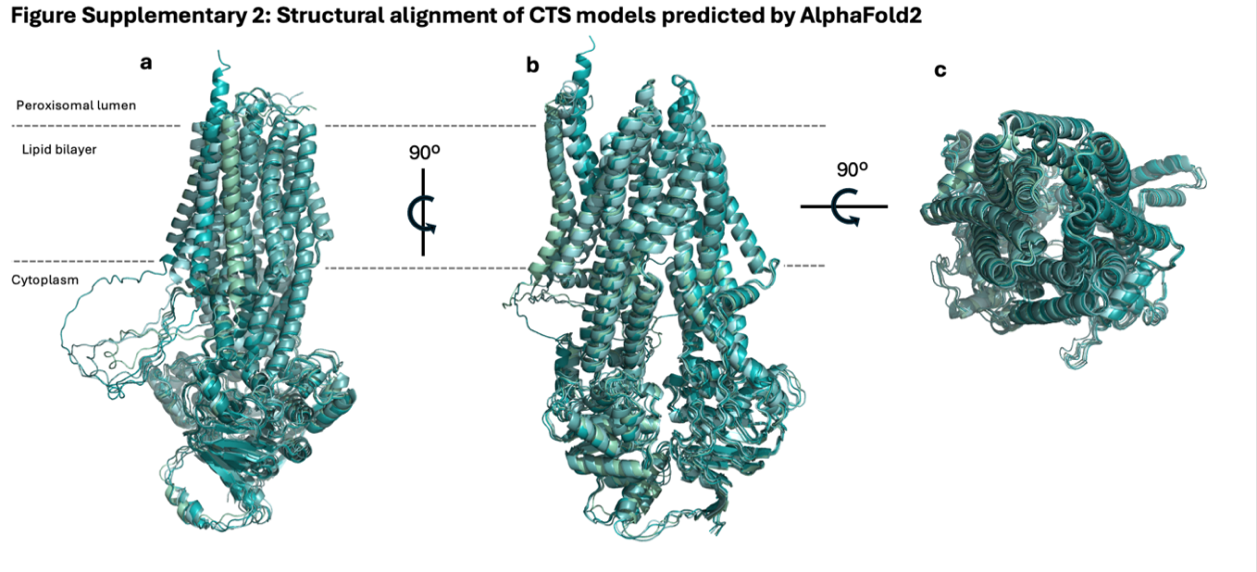
*

***S. Fig 2. a-b*** *Structural alignment of the 5 CTS models produced by AlphaFold3 coloured by different shades of cyan.* ***c*** *Top-view of the structural alignment of the models with the disordered regions removed for clarity.*

***
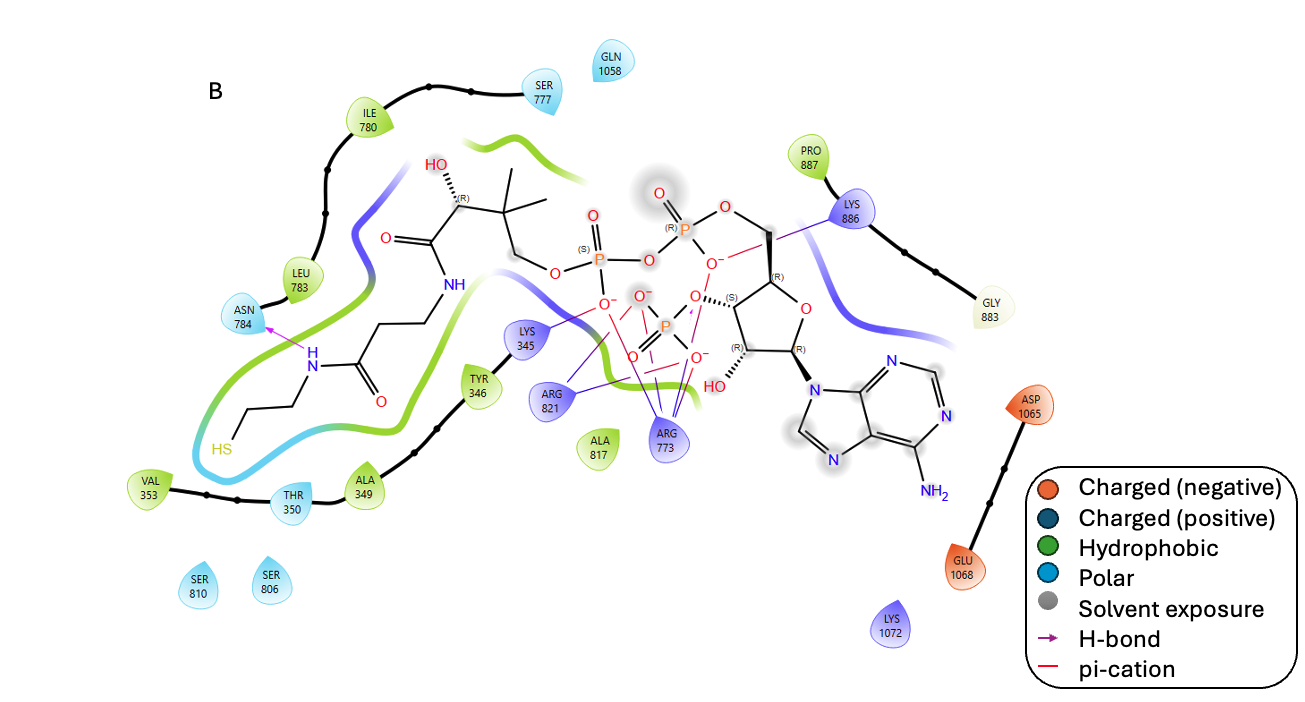
***
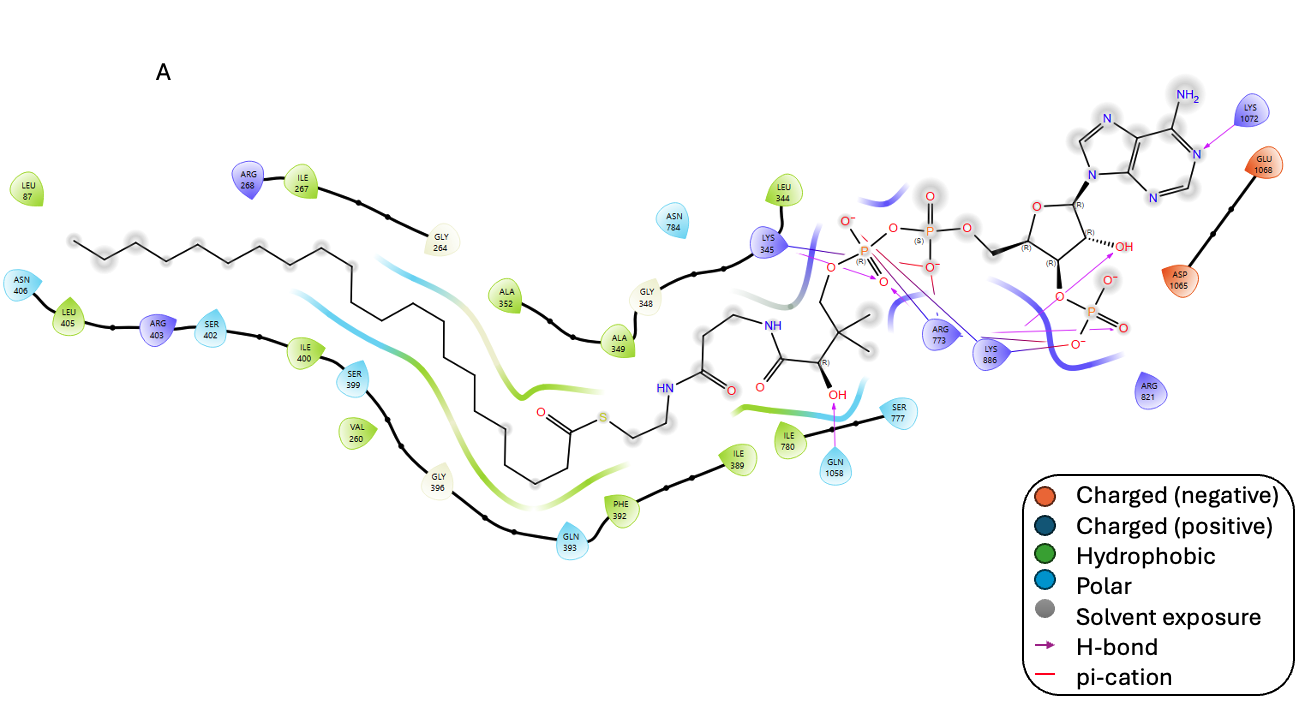


***S. Fig 3****.* ***a*** *Structural alignment of CTS apo model and all available human structures with substrates bound and* ***b*** *top view of the same alignment.*

**

***S. Fig 4. a*** *The residues E300 and E955 found on the coupling helices.* ***b*** *The P350 residue found in NBD1.* ***c*** *Top view of the NBDs with the TMDS removed. The highly conserved residues shown part of the Walker A (K487,K1132) and Walker B (D606,E607 and D1276,E1277) motifs in both NBDs.*

**

***S. Fig 5.*** *Diagram showing the interactions between CTS residues and ligands during docking simulations of a) C22:0-CoA and b) CoA.*


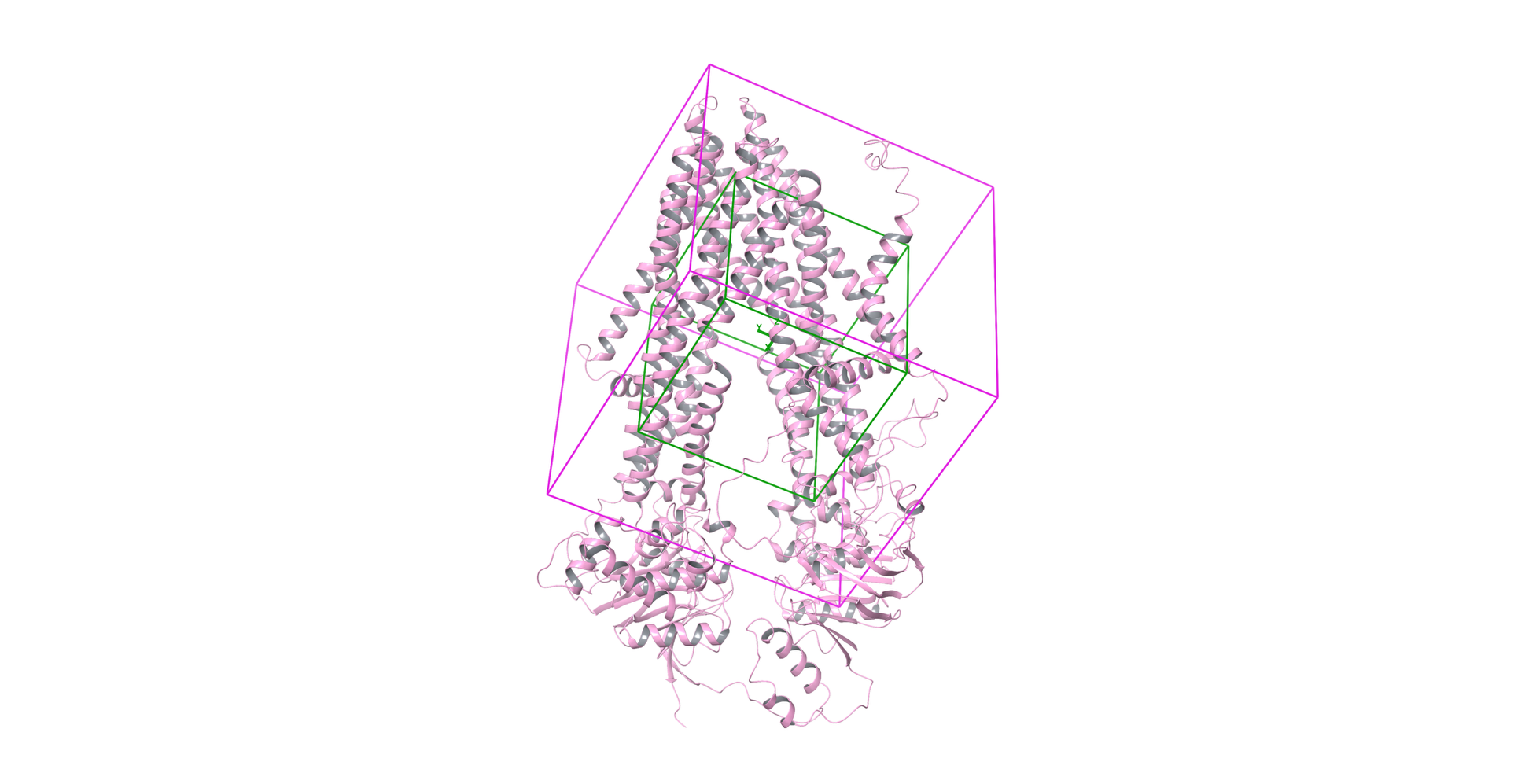


***S. Fig 6.*** *Model of CTS in apo conformation (AlphaFold2) with the grid (66x66x66 Å) used for docking in Glide (Schrödinger).*
